# Artificial Intelligence: Has Its Time Come for Inclusion in Medical School Education? Maybe…Maybe Not

**DOI:** 10.15694/mep.2021.000131.1

**Published:** 2021-05-16

**Authors:** Brandon Ngo, Diep Nguyen, Eric vanSonnenberg

**Affiliations:** 1 University of Arizona College of Medicine - Phoenix

**Keywords:** artificial intelligence, undergraduate medical education, medical students, medical school curriculum, machine learning

## Abstract

This article was migrated. The article was not marked as recommended.

Artificial intelligence (AI) has the potential to strongly modify or even transform the landscape of medicine. Judicious utilization of AI can further propel progress in medical research, facilitate precision medicine, and optimize clinical workflow-the applications are limitless. Although technology and AI algorithms are evolving rapidly and have important implications for future physicians, there is a dearth of literature available for medical students and their educators to learn about AI. While scientific journals provide information regarding AI, they often are written for and by scientists, engineers, and physicians who are well-versed in technology. Currently, medical students must navigate the technical jargon and decipher AI literature without any foundational exposure.

It is difficult for students to understand the implications of AI if they do not have basic knowledge of AI and its current capabilities. A fear about AI is pervasive amongst medical students. There is virtually no literature that provides a fundamental and easily digestible overview of AI for medical students and educators, while also offering suggestions about how to integrate AI into medical school curricula. Our goal is to address the lack of formal AI instruction by presenting an informative primer with curricular suggestions for each medical school year, tailored to medical students and their educators. We seek to present a balanced approach, as there are pros and cons about incorporating AI in undergraduate medical education.

## Introduction

The medical world is undergoing rapid transformation as medical knowledge expands and technology evolves at exponential rates. One defining feature of modern medicine is the fusillade of information. While medical knowledge doubling time was 50 years in 1950, it is now estimated to be only 73 days ( Densen, 2011). This leaves physicians with the seemingly impossible task of synthesizing and organizing the ever-increasing abundance of information.

Recent advancements in artificial intelligence (AI) invite innumerable possibilities to ameliorate the medical information overload ( Wartman and Combs, 2018). The application of AI in medicine is not limited simply to aggregating massive amounts of data. As evidence-based medicine grows to serve the increasingly diverse population through precision and personalized approaches, AI will be an important ally to help define accurate diagnostic and treatment recommendations ( Chen and Asch, 2017; Char *et al.,* 2018). Moreover, AI can be a boon to improve clinical workflow by streamlining onerous administrative tasks, such as endless charting ( Rajkomar *et al.,* 2019). The potential applications of AI in medicine are unlimited, as AI begins to change medicine.

Nonetheless, new technologic advancements driven by AI has brought about uncertainty, and even fear, concerning AI's introduction and integration into the medical field. This paper will focus on the potential impact of AI on medical school education. We will offer curricular suggestions for each undergraduate medical year, and how medical students and curriculum committees can utilize this information immediately, while simultaneously addressing the uncertainties and challenges of integrating AI into medical school education.

Despite the growing significance of AI and its inevitable role in the future of medicine, current undergraduate medical curriculum is largely devoid of information on AI ( Kolachalama and Garg, 2018). The two medical student authors of this AI primer have been surprised by the absence of AI information in their preclinical curriculum, considering their extensive exposure to it as undergraduate engineering majors. Many reasons exist to explain the absence of AI in current medical school education. Given the newness of AI, there likely is a limited number of medical school faculty members who have enough expertise to teach and mentor students on the topic. Furthermore, medical schools constantly are adjusting their curricula to address new and evolving content areas. With no formal requirement for AI in the curriculum by the Licensing Committee on Medical Education (LCME), many schools forgo the topic entirely. Additionally, schools typically choose to focus on the Association of American Medical College's (AAMC) 13 Core Entrustable Professional Activities for Entering Residency (Core EPAs) to prepare students for residency ( Lomis, 2017).

In light of the vast amount of ever-expanding medical knowledge and professional competencies to be covered, it is likely that AI remains an afterthought for most curriculum committees. Medical students are left to fend for themselves, as the lack of curricular exposure to AI leaves them ill-prepared and potentially fearful for the upcoming AI revolution in medicine.

Through this primer on AI, we hope to provide our peers and medical educators with foundational exposure to AI to alleviate apprehension, to provide recommendations on how AI can be integrated into current medical school curricula, and how students and curriculum committees can utilize the opportunities that AI provides. We will also address the barriers and challenges to integrate AI in medical education.

## Pros and Cons of AI in Undergraduate Medical Education

The decision to include AI in medical school curricula is complex, and there are reasonable arguments from both AI proponents and critics. By introducing AI to students early in their medical education, they will be better prepared to handle the ethical, legal, and clinical aspects of AI's future integration into medicine. Today's students will become the future physicians responsible for mediating the union between AI and medical practice. If students can enhance their understanding of AI alongside advancements in AI technology, they can effectively utilize it to provide the best care for patients.

However, incorporating AI into undergraduate medical education is no simple task. Medical school curricula are full of educational requirements including basic science, clinical knowledge, and professional competencies. Currently, there is little to no room to accommodate AI, nor are there many qualified faculty members to teach it. Much of AI's current applications in medicine are hypothetical, thus curriculum committees may not be convinced that AI warrants an immediate place in medical school curricula.

There are numerous pros and cons of including AI in medical school curricula today, and it is important to consider both sides of the argument since there is no clear consensus.

## Glossary of Terms

***Artificial Intelligence (AI)*** *:* A multi-disciplinary field aimed at simulating human intelligence by machines.

***Machine Learning (ML):*** An application of AI in which machines are able to learn without being explicitly programmed to do so. The machines are trained on available data sets to make associations of predictive power ( Panch *et al.,* 2018). For example, ML algorithms have been used to classify cancers from histopathologic images ( Esteva *et al.,* 2017).

**Figure 1:**
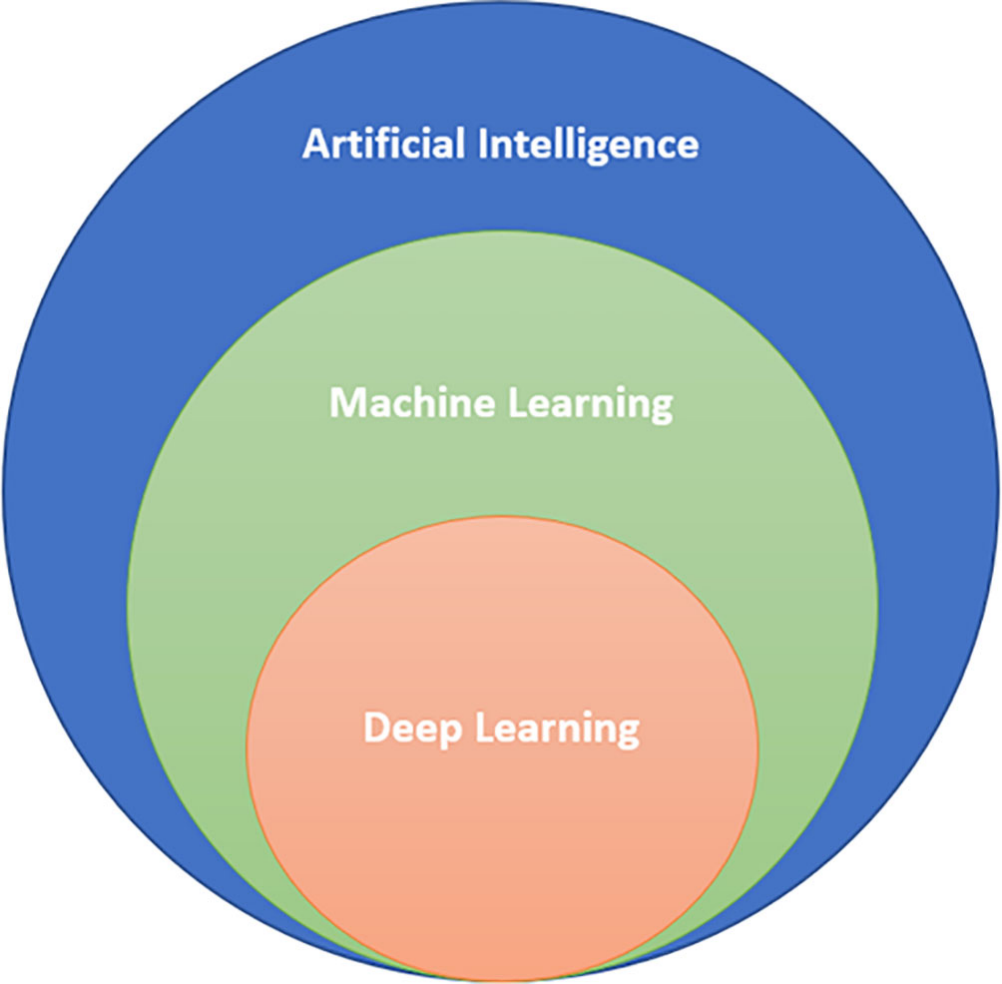
AI/ML/DL Classification.

***Deep learning (DL):*** A subset of ML that utilizes multiple layers of data representation, makes fewer assumptions about the data, and is more computationally efficient at analyzing complex data ( Figure 1). Deep learning methods can be classified as “supervised learning” or “unsupervised learning”.

AI encompasses both ML and DL, with ML being a subset of AI, and DL being a subset of both.

***Supervised learning (SL):*** Maps an input to an output based on a predictive model generated from input-output pairs, provided by a human supervisor. There is an answer key, in that there is prior knowledge of what the output values should be, for the machine to validate its accuracy as it learns from the training set. SL is generally designed for classification (e.g.-determining the type of EKG) and prediction (e.g.-diagnosing myocardial infarction from cardiovascular risk factors).

***Unsupervised learning:*** Parses through an unlabeled dataset and recognizes patterns that are not easily discernible. The training data are unlabeled, and the machine has to extract features and learn inherent structures on its own, without a supervisor or answer key ( Panch *et al.,* 2018). This method is beneficial to analyze heterogeneous, multifactorial diseases with no currently known definitive causes (e.g.-identifying patterns in cancer genomics that are not readily apparent) ( Cheng *et al.,* 2013).

**Figure 2:**
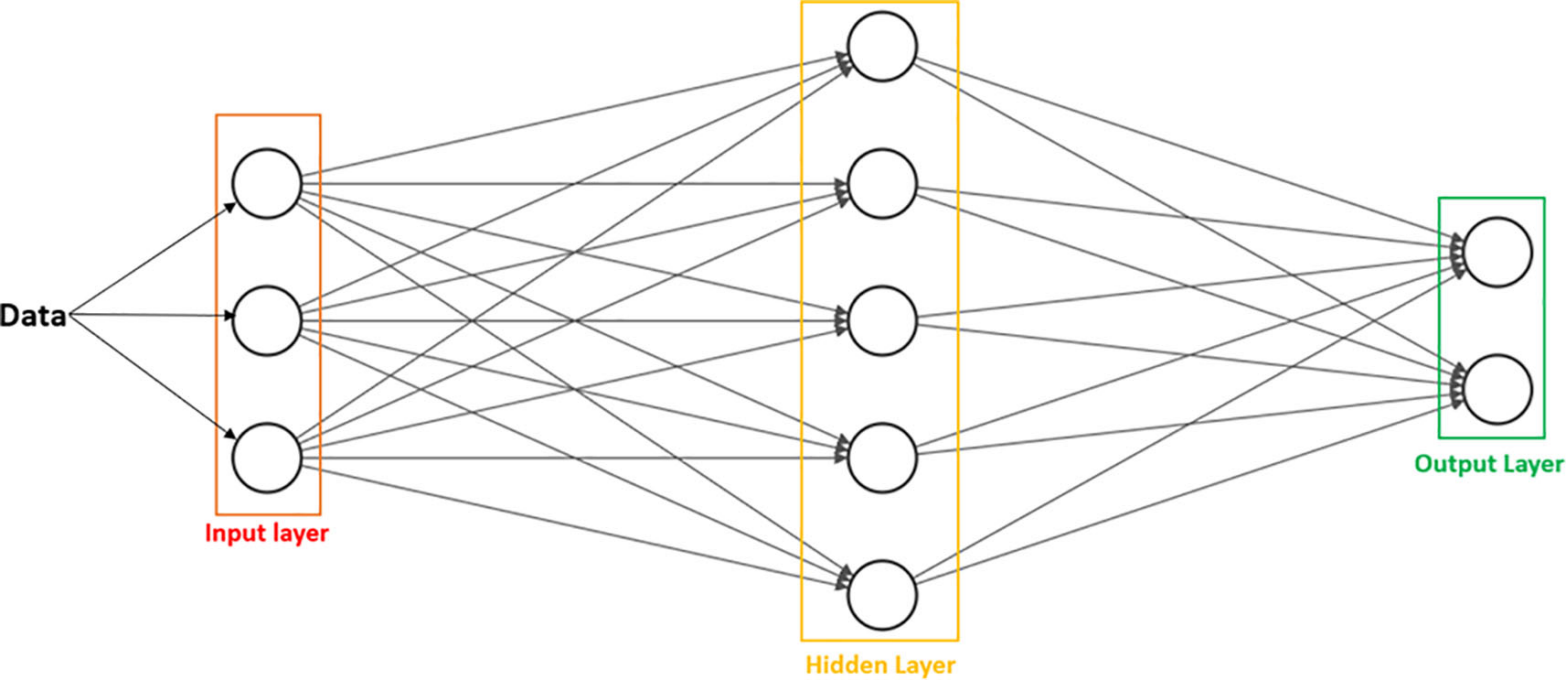
Basic arrangement of ANN.

***Artificial Neural Networks (ANN):*** Are statistical procedures used to arrive at a single output, with the goal of performing a specific task. They are inspired by the information processing ability and organization of neurons in the human brain. ANNs possess nodes, or processing units, that are also called “artificial neurons” ( Scarborough and Somers, 2006). Typically, they are arranged in three major layers: input, hidden, and output ( Figure 2). The *input* layer receives information, while the *hidden* layer processes and derives patterns, ultimately culminating in an *output* that is based on the original information ( Silva *et al.,* 2017). ANNs can learn by supervised or unsupervised learning.

Data are received by the *input* layer, sent to the *hidden* layer for processing, and exported to the *output* layer as answers or analyzed data patterns, depending on the type of problem or task the ANN is designed to solve. The number of nodes is arbitrary and will vary based on parameters set by the user.

### Examples of Supervised and Unsupervised Learning

“Supervised learning” is utilized when we want the computer to emulate human performance on tasks such as classification. One medical example of this would be a computer-generated interpretation of a lung mass on chest x-ray, with the goal of mimicking the diagnostic abilities of a radiologist. The algorithm would need to recognize that a lung mass is present, and then classify whether the abnormality is benign or malignant, based on its previously trained framework.

In contrast, “unsupervised learning” methods are applied when we would like the computer to parse through an unlabeled dataset without an answer key and recognize patterns that are not easily discernible. This ML method is beneficial to analyze heterogeneous, multifactorial diseases with no currently known definitive causes, such cancer ( Cheng *et al.,* 2013). “Unsupervised learning” applied to cancer genomics would be analogous to a research team discovering a cluster of genes linked to numerous, seemingly unrelated cancers that might help explain the pathogenesis.

## Where AI Is, or Isn’t, in Medical School Curricula Today

We developed a Qualtrics survey, approved by the Institutional Review Board at our medical school, to assess the presence or absence of AI in medical school curricula throughout the United States. The survey was distributed via email to 43 current medical students, each from a different U.S. medical school. None of the schools (0/43) surveyed had any AI in their curriculum; however, 4 schools did report having electives and/or extracurricular opportunities that were related to AI.

While AI is still fledgling technology and is not regularly used in clinical practice today, there is potential for it to have significant impact in medicine's future, and medical students may benefit from early introduction of AI in medical school. Undergraduate medical students should have the opportunity for, at the very least, general AI exposure in their education. Such curricular reform would not require in-depth analysis of deep learning algorithms; rather, the goal would be to foster an understanding of general AI concepts and how AI can best be integrated to serve patients and their physicians. This initiative would provide students with a foundation on the topic, allow them to better assess relevant literature, and to prepare themselves for the inevitable changes that AI will bring to the medical field.

## Ethical and Legal Issues

Although AI is a golden opportunity to transform medicine for the better, there are ethical and legal obstacles to consider. If AI algorithms are trained on flawed or incomplete datasets, they likely will generate faulty recommendations that can harm patients. In 2018 for example, IBM Watson's oncology AI algorithm (“Watson for Oncology”) was trained on a non-representative limited dataset, and provided a drug treatment suggestion that would clearly have exacerbated patient bleeding ( Ross and Swetlitz, 2018). A single physician mistake is detrimental to individual patients; an error in an AI algorithm would be catastrophic on a wide scale!

How medical malpractice would be handled in such instances is complex and currently unclear ( Harned *et al.,* 2019). The U.S. Food & Drug Administration (FDA) is in the process of designing a framework for AI software approval in healthcare, and is actively requesting suggestions ( US Food & Drug Administration, 2019). This is the opportune moment for current and future physicians to learn more about AI, and to contribute to the proper implementation of AI in medicine that will be beneficial for patients and physicians.

There has been speculation that AI might widen the gap in healthcare inequality ( Wapner, 2018; Topol, 2019). Several studies have trained algorithms to diagnose melanoma or utilized genomic information and AI to find potential cancer cures, but minorities were vastly underrepresented in the datasets. Without enough minority data in the training sets, the predictions made by the algorithm would be accurate only for non-minority patients.

Additionally, there are significant data security and patient privacy concerns with AI that pose barriers to its implementation ( Brundage *et al.,* 2018). Hackers may be able to gain access to sensitive, patient identifying information or even tamper with AI algorithms to make faulty, malicious suggestions for patient care ( Finlayson *et al.,* 2019). The careful regulation and protection of patient data for use in AI are paramount.

Numerous technology giants, such as Amazon, Google, and Facebook are developing and continuing to implement AI into their businesses. Furthermore, there are hundreds of AI startup companies targeting the healthcare sector ( CBI Insights, 2019). With such growing interest in AI, it is important to be aware of the ramifications of business involvement in the medical field. Although companies might have patient health interest in mind, ultimately they seek financial profit. It is likely that AI algorithms developed by technology companies may find their way into the clinical world. As future physicians who look out for our patients' best interests, it is imperative to help foster this AI transition correctly and respectfully to protect our patients. We medical students and physicians need to be in control of AI's future in medicine, not technology conglomerates!

## The Fear Factor

AI research is increasing in numbers across the various medical specialties, a factor that is important to consider for medical students when choosing their future specialty ( Galimova *et al.,* 2019). But with this new technology comes a level of uncertainty and even fear. The major concern is whether or not AI will replace physicians. The current literature generally emphasizes how AI will be a supplement and a tool for physicians, rather than a substitution ( Krittanawong, 2018). However, a few studies on medical student attitudes have revealed both anxiety and mixed feelings towards AI. One previous study reports how students were discouraged from pursuing the field of Radiology, since they perceived AI as reducing the demand for radiologists ( Gong *et al.,* 2019). Another study reported that medical students favor inclusion of AI in the medical curriculum, and viewed AI as a potentially revolutionizing change for the field of Radiology ( Pinto Dos Santos *et al.,* 2019).

Regardless of where students stand on their views of AI, a survey noted that most understanding by students of AI comes from the media, rather than from formal institutional instruction ( Gallix and Chong, 2019). Although the current trend of research papers is aimed at proving how machines can perform as well, or even better, than clinicians, it should be duly noted that these papers are showcasing how machines are excelling at well-defined tasks that will greatly enhance the efficiency of clinicians ( Buch *et al.,* 2018; Panch *et al.,* 2018). The softer side of medicine-creativity, empathy, and the all-important patient-physician relationship-is less likely, or not at all, to be replaced by AI ( Amisha *et al.,* 2019).

## Areas of Healthcare That AI Could Change: What Medical Students Should Know

There are various areas of healthcare that might be affected by AI in the future. Medical students might benefit from understanding how medicine can be improved through AI, rather than trying to comprehend the intricacies of AI algorithms. The major goal for students would be to appreciate the possible practical and clinical applications of AI.

AI has the potential to impact diagnostics, chronic disease management, selfcare and prevention, clinical decision making, and care delivery ( Topol, 2019). Radiology, Pathology, and Oncology are fields that might be influenced by AI's ability to improve diagnostic accuracy and speed ( Davenport, 2019). However, for AI to be an effective diagnostic tool, it requires enormous patient data training sets that must be anonymized, which could be incredibly costly and difficult to procure.

Managing chronic diseases is no simple task, and it requires that patients regularly follow-up with their primary care physicians. For a multitude of reasons, patients may not be able to physically attend their visits. Digital health management has been suggested as an alternative method to connect physicians and complex patients ( Panch and Goodman, 2016). Additionally, the COVID-19 pandemic has demonstrated the utility of telehealth and its potential for future use ( Hollander and Carr, 2020). While digital health management can help physicians reach more patients, hospitals and clinics will need to decide which patients might benefit most from it. AI, specifically machine learning, has the potential to analyze medical record and insurance data to prioritize patients based on real-time needs, create intervention alerts, and provide follow-up recommendations ( Panch, 2019).

Patients have the capability to manage their own conditions through AI. Utilizing an AI smartphone app, patients can assess skin lesions to detect potential malignancy ( Thissen *et al.,* 2017). AI has shown potential application in mental health as well. An AI-powered chatbot that uses natural language processing has been shown to reduce depression by 28% and anxiety by 18% on average, allowing patients to get real-time support just by texting with their smartphone ( X2 AI, 2020). AI can help empower patients to take control of their health and notify patients when they should see a physician.

One of AI's greatest strengths is its ability to manage increasingly large and complex datasets. Physicians aim to make clinical decisions using evidence-based medicine, and AI can help support this practice. AI algorithms can generate real-time predictions to suggest optimal patient treatment that can augment a clinician's capacity to provide effective and personalized care ( Garcia-Vidal *et al.,* 2019). Additionally, AI has the potential to streamline clinical workflow, reducing administrative tasks, such as clinical note taking and navigating convoluted electronic health records (Reddy, Fox and Purohit, 2018). The proper implementation of AI can transform healthcare delivery by improving efficiency, and by allowing physicians to provide more and better care to more patients ( Spatharou, Hieronimus and Jenkins, 2020).

## General Recommendations for Those Students Who Are Interested in AI

Medical students around the world can develop numerous skills during their training that will aid them in learning about AI, regardless of curricular design. We encourage students to develop AI literacy by starting with general articles, podcasts, or videos that provide a fundamental overview of AI. Once students feel more comfortable with the terminology, they can seek more complex sources of AI information that incorporate medical applications. While reading and learning more about AI, we suggest that students continue using their critical thinking and problem-solving skills by asking why certain AI/ML methods are used in different situations. For example, when we want to mimic the diagnostic capabilities of a pathologist, we would prefer to utilize supervised learning instead of unsupervised learning, because we can train the algorithm with the same set of slides that pathologists use to hone their skills.

Learning about AI should be engaging, and we advise interested students to use their own backgrounds in either computer science, engineering, ethics, law, or more to amplify their learning experience. Whether that be delving into the technical aspects of algorithms or furthering the discussion of how to handle patient data privacy when using AI, there are numerous opportunities to get involved. Additionally, medical students can get hands-on experience with AI by establishing structured collaborations between medical schools and engineering schools. AI is still in its infancy and currently is not ready for full implementation into the medical field. However, when that time comes, it will be future physicians (i.e. - today's medical students) who will lead the charge to incorporate AI into clinical practice.

## General Recommendations for Students Who Are NOT Interested in AI

AI may not be an appealing topic to all medical students. Whether or not to integrate AI into medical school curricula remains a controversial topic. However, some students will find current applications of AI in medicine rather intriguing. These students may benefit most from faculty-and student-led demonstrations of AI's capabilities in clinical decision making, diagnostics, and workflow. They can be shown AI-driven radiographic image interpretations, analyses of histologic slides from the pathology lab, and breast cancer detection. Beyond this, students might not see much utility in learning about AI in depth. We encourage these students to keep an open mind throughout their training and to be aware of AI, but not necessarily delve into details.

## Curricular and Student Recommendations Throughout the Four Years of Medical School

The authors are from the U.S., and offer specific suggestions on how to integrate AI during each year of training, based on the U.S. medical school model. While the organization of this section might be framed from a U.S. perspective, the provided suggestions can be applied broadly.

### For Schools That Want to Include AI in Their Curricula Officially

#### MS1

First year medical students would benefit from an introductory lecture that addresses the basics of AI-what it is, basic terminology, how it is and will be integrated into modern medicine. Currently, very few schools are taking this approach by incorporating introductory lectures ( Kolachalama and Garg, 2018). Medical educators can develop a crash course in essential knowledge that is needed to understand the basics of AI.

Considering the increasing amount of AI research done across the various specialties, the fear and anxiety towards AI should be addressed early ( Park *et al.,* 2019). Primer articles such as this, and other introductory AI papers will be helpful to provide students a more informed view on AI in medicine, rather than gaining information from hearsay and the media ( Kohlert *et al.,* 2017; Chan and Zary, 2019). Finally, interested students should be introduced to the trends in medical AI research, and then potentially seek research mentors who might specialize in AI medicine. Medical students who become knowledgeable and involved with AI research undoubtedly will be viewed favorably and up-to-date.

#### MS2

With the aid of nearby engineering institutions, medical educators can form interdisciplinary innovation groups that create AI projects to solve novel challenges. These groups would bring medical and engineering students together, to help bridge the gap between medicine and technology. For medical educators, there are various papers online that provide a tutorial on building an AI algorithm to be used on free public data ( Jiang *et al.,* 2017; Sidey-Gibbons and Sidey-Gibbons, 2019). In constructing these algorithms, students will begin to develop an understanding of how to phrase problems that are amenable to AI solutions. We suggest that lecturers work on these mini-projects together in person with the students, instead of creating extra homework assignments or an online module.

#### MS3

Medical educators can prepare medical students by training them to share AI articles that relate to patients they see. Students can bring so much value to their patients and fellow colleagues by sharing new methods and information gleaned from AI to improve patient care. Faculty attendings likely will be appreciative by learning the value of AI from medical students.

#### MS4

Fourth year schedules are highly variable, depending on the medical school's requirements and the students' desires. Creating an elective course that enables students to design their own AI module or update an existing lecture may benefit students who are interested in medical education ( Masters, 2019). With residency interviews on the horizon, medical educators can best support their students by helping them incorporate their AI experiences into their CV. Students should be coached on how to best present themselves as future pioneers in AI medicine that will be a notable point of distinction in residency interviews, regardless of the medical specialty. Medical students with an AI background have enormous potential to help residency programs create educational resources for AI medicine.

### For Schools That DO NOT Want to Include AI in Their Curricula Officially

#### MS1

Creating interest groups centered around AI medicine can be a productive option for interested students. During medical school, students contemplate which specialties to enter, and now AI can help their decisions. Medical students with undergraduate majors in engineering can utilize their training to take advantage of AI opportunities. Students can utilize this opportunity to develop their research skills in AI, as well as contribute to an evolving field. As their AI knowledge grows, they can develop their own instructional modules for fellow classmates to help them understand the basics of AI.

#### MS2

Medical students can further advance their AI knowledge through reading targeted research articles or following journals that specialize in AI medicine. Appendices 1 and 2 are lists of review articles that give a broad overview of the various topics in AI medicine, as well as a list of journals that focus on AI medicine. We encourage medical students to use these appendices as primers to create their own journal clubs. Developing an aptitude for scrutinizing and interpreting AI articles will open doors for engaging research. Experienced medical students will become more comfortable to educate patients and physicians alike. Also, the students can facilitate communication between engineers from their colleges and clinical physicians ( Johnston *et al.,* 2019).

There are many startup companies today that are aiming to integrate their AI innovation into the existing healthcare workflow; medical students who are knowledgeable about AI have a golden opportunity to mediate a working relationship between their academic institution and these companies ( Barnett *et al.,* 2019).

#### MS3

By this time, medical students are preparing to enter the wards and it would be helpful for them to be aware of possible AI applications that they may encounter in clinical practice. An AAMC News article reports that 40% of Internal Medicine teaching programs utilize the “Human Dx Project”, an AI project that collects and tracks clinical reasoning data from clinicians globally in an attempt to build an accurate, evolving diagnostic tool ( Gong *et al.,* 2019). Students have the potential to use this tool to enhance their clinical reasoning, and to contribute case reports to the collective diagnostic repository. Additionally, students on clerkships will be able to see first-hand what clinical areas might benefit most from AI.

#### MS4

Medical students will be preparing for residency interviews and might benefit from reflecting on their personal experiences with AI. They may select programs that will allow them to further explore AI in residency and present themselves as trailblazers for AI in medical education. Regardless of the specialty, students who have a background in AI can add value to their intended medical field of practice.

## What to Substitute in the Curriculum for AI

Medical school curricula are filled to the brim with basic science, clinical skills, and professional competency requirements. Removing anything from the current curricula is no easy feat, and doing so for AI currently might not be feasible. However, with the United States Medical Licensing Examination (USMLE) Step 1 becoming pass/fail in 2022, there should be more flexibility in the pre-clinical curricula to insert AI.

## Conclusion

We encourage medical students to dive in and explore the rapidly evolving field of AI by reading research articles, watching educational videos, having thought-provoking conversations with peers and faculty, creating AI interest groups, and/or using any method that is convenient and comfortable for them. Medical educators will be playing a prominent role in structuring medical education about AI. By understanding how AI might impact healthcare and preparing ourselves to utilize AI, we can lessen the fear and anxiety surrounding the topic. We must keep in mind that the foundation of medicine resides in the patient-physician relationship, something that AI can help enhance, but should not, and cannot, replace. AI will be our ally in the future of medicine, helping us to achieve our ultimate goal of best serving our patients. There is no better time than now to begin cultivating this relationship.

## Take Home Messages

• AI is a blossoming field that will have important implications for medicine.
• A fundamental understanding of AI and its possible applications in medicine are crucial for proper implementation into clinical practice.
• Early medical student exposure to AI, with support from medical educators, can help reduce the anxiety, fear, and enigma surrounding AI.
• While AI can have positive ramifications for the medical field, it is not infallible and there are complex issues to consider regarding its use.
• Medical students can expand on their knowledge of AI throughout their training and careers to facilitate ethical and effective use of AI in healthcare.

## Notes On Contributors

**Brandon Ngo** is a third-year medical student at the University of Arizona College of Medicine - Phoenix. ORCID ID: https://orcid.org/0000-0002-3246-7591

**Diep Nguyen** is a third-year medical student at the University of Arizona College of Medicine - Phoenix.

**Eric vanSonnenberg,** MD, is a Professor at the University of Arizona College of Medicine - Phoenix.

## Figures and Tables

**Appendix 1:** A list of journals that focus on AI medicine. Inclusion criterion is medically-oriented AI journals, as opposed to strictly engineering or highly technical AI journals.

Journal Name	URL Link
Radiology: Artificial Intelligence - Radiological Society of North America (RSNA)	https://pubs.rsna.org/journal/ai
Artificial Intelligence in Medicine - ScienceDirect	https://www.sciencedirect.com/journal/artificial-intelligence-in-medicine
Journal of Medical Artificial Intelligence (JMAI)	http://jmai.amegroups.com/
Nature Medicine	https://www.nature.com/nm/
American Medical Association (AMA) Journal of Ethics	https://journalofethics.ama-assn.org/issue/artificial-intelligence-health-care
New England Journal of Medicine (NEJM)	https://www.nejm.org/
Journal of Medical Internet Research (JMIR)	https://www.jmir.org/
British Journal of General Practice (BJRP)	https://bjgp.org/
Clinical Informatics | Yearbook of Medical Informatics - Thieme	https://www.thieme.com/books-main/clinical-informatics/product/4709-yearbook-of-medical-informatics
Journal of the American College of Radiology (JACR)	https://www.jacr.org/

**Appendix 2:** A list of review articles that give a general introduction to AI medicine.

Topic	URL Links
History of AI in medicine	Artificial Intelligence In Healthcare: Past, Present and Future https://svn.bmj.com/content/2/4/230
Safety of AI	Artificial Intelligence, Bias and Clinical safety https://www.ncbi.nlm.nih.gov/pmc/articles/PMC6560460/
Examples of AI performing well	Detecting Asymptomatic Left Ventricular Dysfunction from EKG's https://www.nature.com/articles/s41591-018-0240-2
Commentary on AI in Medicine	AI in Health Informatics: Hype or Reality? https://www.ncbi.nlm.nih.gov/pmc/articles/PMC6697542/
