# Supplementary material for: Artificial Intelligence: Has Its Time Come for Inclusion in Medical School Education? Maybe…Maybe Not
Source: MedEdPublish (2016). 2021 Sep 3;10:131. Originally published 2021 May 16. [Version 2] doi: 10.15694/mep.2021.000131.2 (PMC10939546; doi:10.15694/mep.2021.000131.2)
Supplement: Peer review history

# Reviewer report: Reviewer response for version 2

**DOI:** 10.21956/mep.20212.r31295

**Published:** 2021-10-12

**Authors:** Julie Hunt

**Affiliations:** 1 Lincoln Memorial University

This review has been migrated. The reviewer awarded 4 stars out of 5 This personal opinion piece, written by two medical students and a medical educator, makes a case for including learning about AI and medicine during students' training in order to introduce them to the topic, which is expected to grow in the future and further impact the face of medicine. The authors provide suggestions for inclusion in the medical school curriculum and extracurricular options, though they do not directly address how some of the more time-consuming options (e.g. Year 2 - finding time for the projects suggested) might fit into the curriculum, which is already very full. I would also have appreciated a discussion of how medical educators could become qualified to teach about AI in medicine, as the authors acknowledge that most educators lack experience in the field.The order of how things were presented could have been more reader-friendly. For example, AI should be defined early in the introduction, and instead the reader has to read quite far into the article to get a working definition. Further, Appendices 1 and 2 should be referenced earlier in the text, when the authors first mention that students should review fundamental articles. I appreciate the inclusion of the appendices, as these can lead students and educators to additional information.I think this opinion piece may interest those who view AI as an inevitable improvement to medicine and who look for a way to incorporate it into the medical school curriculum, or simply to educate themselves about AI in medicine.

Reviewer Expertise:

NA

No decision status is available

# Reviewer report: Reviewer response for version 2

**DOI:** 10.21956/mep.20212.r31294

**Published:** 2021-09-15

**Authors:** Ken Masters

**Affiliations:** 1 Sultan Qaboos University

This review has been migrated. The reviewer awarded 5 stars out of 5 I am pleased to see that the authors have addressed the issues that I raised in my review of Version 1. Although I would like to have seen more concrete examples of AI in the curriculum, this is a useful opinion piece for medical schools to begin conversations about how to introduce AI into their curriculum, while simultaneously being aware of some of the difficulties of doing so. Well done.

Reviewer Expertise:

NA

No decision status is available

# Reviewer report: Reviewer response for version 1

**DOI:** 10.21956/mep.19017.r27294

**Published:** 2021-05-31

**Authors:** Daniel Salcedo

**Affiliations:** 1 Chiba University Hospital

This review has been migrated. The reviewer awarded 3 stars out of 5 The status of AI integration into the medical curriculum and the need for AI competency as a future skill for care providers is an important area of discussion.I applaud the interest of the authors in this area, and even though they make a compelling argument, there are some points of view missing from this discussion.The first of these is the "Black Box" concept, in which early attempts to introduce AI to clinical practice have met with heavy resistance from practitioners (such as occurred with Watson). Physicians are, in many cases, expected to "trust" the suggestions of an AI without a clear understanding of how the system used data to produce recommendations. The "Fear Factor" mentioned in this article is less related to job security than ensuring patient safety. Similarly, we would hesitate to recommend a drug without first understanding how it works and seeing controlled studies demonstrating its effectiveness and safety.The next consideration that needs to be included in this paper is about who can teach AI-enhanced practice. There are very few medical educators with enough AI competency to teach medical students best practices in its application to clinical care. And while non-medical AI specialists may teach basic concepts of AI to medical students, its actual integration into practice is more challenging to teach since non-medical educators might have difficulty understanding how AI is actually applied at the point of care. Any plan to introduce AI into the medical curriculum must include first the training of teaching faculty. Until a sufficient amount of AI-competent faculty is developed, it is impossible to consider a formal integration into the medical curriculum.I really enjoy the fact that medical students were motivated enough to write this article. Still, I think it is necessary to also obtain perspectives from practicing physicians, faculty, and policymakers to provide a more realistic and well-rounded discussion of this important subject. I would like to encourage you to discuss the subject with different stakeholders and include a more in-depth analysis.

Reviewer Expertise:

NA

No decision status is available

# Reviewer report: Reviewer response for version 1

**DOI:** 10.21956/mep.19017.r27295

**Published:** 2021-05-19

**Authors:** Ken Masters

**Affiliations:** 1 Sultan Qaboos University

This review has been migrated. The reviewer awarded 3 stars out of 5 An interesting piece on AI in medical school curricula. The paper begins well by discussing uncertainties and potentials opened by AI in medicine, and the importance of ensuring the medical students are familiar with AI concepts. The glossary is useful. The authors also propose some aspects of AI that could be taught to medical students. Some issues need addressing:• The paper should clarify that it discusses the US schools only (Although this is stated later in the paper, it should be made sooner). E.g. statements like “With no formal requirement...forgo the topic entirely” should indicate that this applies to the US only. Other instances exist; I recommend the authors go through their paper, correcting any misconceptions about the applicability of their arguments.• Unfortunately, the first paragraph (and possibly the second) under the heading “Where AI Is...Today” should be removed. This survey was distributed to 43 students, with no details (when/how conducted, who the students are, how selected, etc.), so this undermines the paper. Either the authors should report in detail on this survey or not at all.• If the second paragraph is retained, “While AI is...not regularly used in clinical practice…” should be supported by the literature. This will be difficult, as “regularly used” is vague, and multiple papers show AI being used in clinical practice.• “Patients have the capability...through AI.” It would strengthen the paper if the authors could relate these comments about patients to the concept of e-patients’ using medical systems, the need to teach medical students about e-patients, and how to work with them, as e-patient power will grow as AI systems become more accessible to them.• Although the curriculum is interesting, the ideas seem presented as a mostly stand-alone course, with little reference to anything else that the students are studying. So, when the students are studying AI, there is little contextual input from what else they are studying at those levels.o If the authors wish it to be so, they really should explain why this is a preferred way of teaching something that will become so deeply embedded into every aspect of medical practice. This is also important because the Abstract recognises the need to “integrate AI into medical school curricula”.o If the authors do not wish it to be stand-alone, then the paper would be strengthened if other contextual topics were discussed, so, where readers’ curricula differed, the AI curriculum could be adjusted. (This is also important because the authors argue that the course could be applied outside the US). As it stands, adjusting the AI course might be possible, but, to do so would require knowledge of what is covered at the different levels.• In both cases, more detail is required on content, especially on teaching the practical use of systems that currently utilise AI. Otherwise, one is left with the feeling (echoed by the Conclusions’ exhortation for medical students to “dive in..by reading research articles”) that little more than a reading articles is required.• A personal preference: The authors may reconsider their suggestion that AI should be introduced through lectures focusing on the terminology of AI. AI is the most exciting development in computing history, and will have an astounding impact on medical practice. We should not recommend that medical students’ first exposure to AI should be through lectures (I can imagine Lecture 1 entitled “The terminology of AI”, in which lists of AI definitions and descriptions with examples are given to be rote-learnt – and students bored to death.) I'm sure the authors know of better ways to introduce AI than through lectures. Perhaps they could consider some of these; if not, then rather remove reference to lectures entirely, and leave it up to the readers’ imagination. (Poorly-resourced institutions may have to resort to lectures, but I would not like to see this as a recommendation).• “However, with the United States Medical Licensing Examination...to insert AI.” This requires support from the literature, explaining how the change will grant greater flexibility, and how it will allow for easier inclusion of an AI course into the curriculum.So, while I like that the authors have highlighted AI's importance, and that it should be introduced to medical students, the stand-alone nature of what is portrayed in the paper concerns me, and lowers its value. I would like to see Version 2 of the paper in which this and the other issues raised are addressed.

Reviewer Expertise:

NA

No decision status is available
